# Morphological plasticity and visual acuity in the natural course of epiretinal membrane-foveoschisis: A longitudinal OCT study

**DOI:** 10.1038/s41433-026-04304-8

**Published:** 2026-02-17

**Authors:** Annegret Hetzel, Caroline J. Wenzel, Faik Gelisken, Kübra Atay Dinçer, Daniel A. Wenzel

**Affiliations:** 1 Department of Ophthalmology, University of Tübingen, Tübingen, Germany; 2 Department of Ophthalmology, Eskişehir Osmangazi University, Eskişehir, Turkey

**Keywords:** Retinal diseases, Predictive markers

## Abstract

**Objectives:**

To characterise the natural course of epiretinal membrane-foveoschisis (ERM-F) using optical coherence tomography (OCT) and analyse morphological and visual changes.

**Methods:**

Retrospective single-centre study of treatment-naïve idiopathic ERM-F eyes. Functional and morphological changes from baseline to the final visit were assessed. The primary outcome was change in best-corrected visual acuity (BCVA). Secondary outcomes included subtype transitions, progression to lamellar macular hole (LMH) or mixed ERM-F/LMH.

**Results:**

A total of 124 eyes from 113 patients (mean age 67.9 ± 8.0 years) were followed for 58.8 ± 47.4 months. Mean BCVA remained stable (baseline: 0.16 ± 0.15 logMAR; final: 0.18 ± 0.15 logMAR; *p* = 0.29). Baseline ERM-F subtypes were open-flat (61.3%), open-elevated (24.2%), and closed (14.5%), shifting to 46.8%, 28.2%, and 9.7%, respectively, at the final visit. Conversion to mixed ERM-F/LMH and LMH occurred in 11.3% and 2.4% of eyes, respectively; two eyes (1.6%) developed a normal foveal contour, while two eyes progressed from open-flat ERM-F via mixed ERM-F/LMH phenotype to full-thickness macular hole. Central foveal thickness (CFT) did not change significantly (*p* = 0.457). Structural changes included increased foveal wall verticalisation and emergence of subfoveal EZ disruption, undermined edges, retinal tissue loss, and foveal bump in a subset of patients. Subfoveal EZ disruption was associated with worse final BCVA on univariable analysis, but not after multivariable adjustment for baseline BCVA and cataract surgery.

**Conclusion:**

ERM-F shows morphological plasticity with generally stable visual outcomes. A minority of eyes progress to mixed ERM-F/LMH or LMH.

## Introduction

Epiretinal membrane-foveoschisis (ERM-F) is a vitreomacular interface disorder characterised by the presence of a contractile epiretinal membrane (ERM) exerting tangential traction, resulting in schitic changes in the Henle fibre layer (HFL) and disruption of retinal architecture [1–3]. Additional findings frequently include intraretinal cystoid spaces, increased retinal thickness, and retinal wrinkling [1, 4, 5]. In contrast to lamellar macular hole (LMH), ERM-F is primarily tractional and typically lacks significant retinal tissue loss. Formerly, LMH was divided into tractional and degenerative types, with the tractional subtype now reclassified as ERM-F for eyes presenting with tractional ERM and associated foveoschisis [6]. Recently, an international consortium established standardised, consensus-based definitions of ERM-F, LMH, and macular pseudohole (MPH), providing clarity and uniformity in diagnosis [1].

Despite increasing recognition of ERM-F as a distinct clinical entity, its natural history remains incompletely characterised. While some patients exhibit morphological and functional stability, others progress to more advanced structural changes, including enlargement of foveoschisis and the emergence of LMH-related features accompanied by visual decline and metamorphopsia [3, 7]. Although treatment options range from watchful observation to macular surgery, data on the natural progression of treatment-naïve ERM-F eyes are limited [3, 4, 8]. Predicting progression remains a clinical challenge, and the identification of factors influencing the natural course may enhance disease monitoring and management.

This study aims to characterise the visual outcomes and longitudinal morphological changes of treatment-naïve ERM-F eyes using OCT.

## Methods

This retrospective longitudinal cohort study included patients diagnosed with treatment-naïve idiopathic ERM-F. Diagnosis was established according to the international consensus-based optical coherence tomography (OCT) criteria for ERM-F [1]. Inclusion criteria were: (1) presence of ERM with foveoschisis at the level of the HFL; (2) a minimum follow-up duration of six months; and (3) available OCT examinations at baseline and final follow-up. Exclusion criteria included: (1) secondary ERM (e.g., prior intraocular inflammation, tractional peripheral retinal breaks, retinal detachment, retinal laser photocoagulation, full-thickness macular hole (FTMH), or ocular trauma); (2) high myopia (spherical equivalent ≤ -6 dioptres); (3) previous intraocular surgery other than uncomplicated cataract surgery; (4) coexisting macular diseases; (5) any mandatory OCT features of LMH defined by Hubschman et al. [1]; (6) eyes with vitreofoveal adhesion defined as posterior vitreous detachment (PVD) stage 0-1 (only stage ≥ 2 were included) [9]; or (7) stellate non-hereditary maculoschisis or X-linked retinoschisis.

Ophthalmologic examination included best-corrected visual acuity (BCVA), slit-lamp examination, intraocular pressure measurement, dilated fundoscopy, and spectral-domain OCT (SD-OCT; Heidelberg Spectralis, Heidelberg Engineering GmbH, Heidelberg, Germany) of the macula. Snellen BCVA values were converted to logarithm of the minimum angle of resolution (logMAR) for statistical analyses. OCT acquisition followed a standardised protocol comprising a 20° x 15° macular volume scan, high-resolution foveal radial scans, and two perpendicular cross-sections through the optic disc.

The primary outcome was the change in BCVA (logMAR) between baseline and final visit. Change in BCVA was defined as final BCVA minus baseline BCVA (ΔBCVA = final BCVA – baseline BCVA); positive values indicate worsening and negative values indicate improvement. A clinically meaningful difference in BCVA was defined as ≥0.1 logMAR, corresponding to approximately one Snellen line. Given the retrospective design, no a priori sample size calculation was performed. Post hoc, the available sample size (124 eyes) provided adequate power to detect a clinically meaningful change in BCVA of 0.1 logMAR. Secondary outcomes included: (1) the development of any mandatory features for LMH, and (2) change in ERM-F subtype. Eyes that developed a FTMH during follow-up were analysed descriptively as a separate terminal outcome. For ERM-F natural history analyses, these eyes were censored at the last visit prior to FTMH diagnosis and were not included as a final ERM-F subtype. Additionally, structural parameters including central foveal thickness (CFT), horizontal schisis diameter, microcystoid spaces in the inner nuclear layer (INL), retinal wrinkling, foveal wall verticalisation, epiretinal proliferation (EP), subfoveal ellipsoid zone (EZ) disruption, and foveal bump were analysed.

**Fig. 1:**
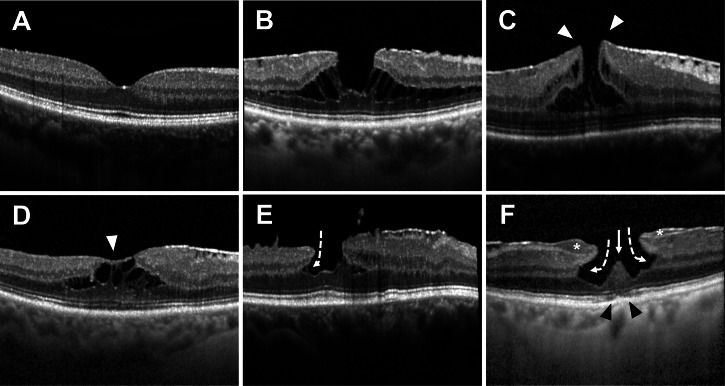
Representative examples of morphological phenotypes of epiretinal membrane foveoschisis (ERM-F), lamellar macular hole (LMH) and mixed ERM-F/LMH graded in this study. **A** Normal foveal contour. **B** Open-flat ERM-F. **C** Open-elevated ERM-F, with foveal edges elevated above the surrounding retinal plane (white arrowheads). **D** Closed ERM-F with epiretinal membrane covering the fovea (white arrowhead). **E** Mixed ERM-F/LMH showing schisis, undermined edge (dashed arrow), and irregular foveal contour. **F** LMH with undermined edges and retinal tissue loss (dashed arrows), irregular foveal contour, epiretinal proliferation (star), foveal bump (white arrow), and ellipsoid zone disruption (black arrowheads).

ERM-F was classified as “open” or “closed” based on foveal ERM coverage [5]. Open subtypes were further classified as “flat” (foveal borders at or below the level of the adjacent retina) or “elevated” (foveal edges elevated above the surrounding retina). Mixed ERM-F/LMH was defined as ERM-F with the presence of at least one mandatory LMH-associated feature indicating tissue alteration (retinal tissue loss and/or undermined foveal edges), while still fulfilling the diagnostic criteria for ERM-F. Irregular contour alone was not sufficient for mixed ERM-F/LMH subtype classification. This category reflects a transitional phenotype between tractional ERM-F and fully developed LMH. Conversion to LMH was defined as the development of all mandatory OCT criteria for LMH in the absence of foveoschisis. Mandatory features for LMH were: (1) irregular foveal contour, (2) retinal tissue loss, and (3) undermining of the foveal edges (inner retinal angle < 90°) [1]. Exemplary OCT scans of the classified subtypes are shown in Fig. 1.

Central foveal thickness (CFT) was defined as the distance from the inner limiting membrane to the inner border of the retinal pigment epithelium (RPE)/Bruch’s complex, measured manually on the foveal OCT B-scan. The horizontal schisis diameter was defined as the maximal extent of the foveoschitic cavity, measured on the horizontal foveal OCT B-scan as the distance between the outermost points of the schisis. All measurements were performed manually using the digital calliper function of the Heidelberg Eye Explorer software (Version 2.5.9; Heidelberg Engineering GmbH, Heidelberg, Germany). OCT images were graded by experienced readers using standardised definitions based on published consensus criteria and the subtype classification [1, 5]. Subtype grading at baseline and final visit was independently evaluated by two masked graders. Baseline ERM-F morphology was classified into three subtypes: open-flat, open-elevated, and closed. At the final visit, six categories were used: open-elevated, open-flat, closed, mixed-type ERM-F/LMH, LMH, and normal foveal morphology. Intergrader agreement was assessed using percent agreement and Cohen’s kappa statistic for nominal categories. Cohen’s kappa was calculated exclusively based on the independent assessments of the graders. In cases of disagreement, a consensus decision was reached through joint review, and the consensus classification was used for all subsequent analyses. Manual quantitative measurements were performed by a single grader.

INL microcystoid spaces were defined as small, round, or elliptical hyporeflective areas distinct from the vertically oriented schitic spaces in the HFL of ERM-F. Retinal wrinkling refers to undulating folds or traction lines on the retinal surface caused by ERM contraction. EP was defined as a preretinal structure adjacent to the internal limiting membrane that appears homogeneous and isoreflective and can clearly be distinguished from thinner hyperreflective ERM. Subfoveal EZ disruption was considered when an interruption or loss of that structure was found. A localised elevation in the fovea seen on OCT was considered a foveal bump. Foveal wall verticalisation was defined as a markedly vertical slope of the foveal pit [1]. All assessed morphological parameters defined above are illustrated in Supplementary Fig. S1.

Posterior vitreous detachment (PVD) was assessed using OCT based on the visibility and position of the posterior hyaloid membrane relative to the macula. PVD, indicating incomplete or complete vitreous detachment, was diagnosed if the posterior hyaloid was visibly detached or seen as a hyperreflective line above the macula (stage 2 or higher) [9].

All statistical analyses were performed using JMP version 18.0.2 (JMP Statistical Discovery, Cary, NC, USA). Continuous variables were summarised as mean ± standard deviation (SD). For variables with skewed distributions, results are additionally reported as median with interquartile range (IQR). Categorical variables were expressed as absolute numbers and percentages. Normality was assessed using the Shapiro-Wilk test. Paired continuous variables were compared using the paired t-test or Wilcoxon signed-rank test, as appropriate. Comparison of continuous variables between more than two independent groups was performed using Kruskal-Wallis test, followed by Dunn’s post hoc test for pairwise comparisons when appropriate. Paired categorical variables were compared using McNemar’s test; Bowker’s test of symmetry was used where appropriate. To analyse morphological progression, contingency tables were used to assess changes in ERM-F subtypes and other morphological parameters over time. Associations between morphological parameters and visual outcomes were explored using univariable and multivariable regression models. Univariable analyses of multiple OCT features were exploratory in nature. All predictors were first screened using univariable linear regression for both final BCVA and ΔBCVA. Variables with *p* < 0.10 and/or strong biological plausibility were entered into multivariable models. All tests were two-sided. Statistical significance was defined as *p* ≤ 0.05 for all tests.

The study was approved by the institutional ethics committee of Eberhard Karls University Tübingen (reference number: 177/2020BO2) and conducted in accordance with the Declaration of Helsinki.

## Results

A total of 124 eyes from 113 patients with idiopathic ERM-F were included. Eleven patients (9.7%) had bilateral disease. The mean age was 67.9 ± 8.0 years (range 46-83 years), and 73.5% (83/113) were female. The mean age did not differ between sexes. The mean follow-up period was 58.8 ± 47.4 months (range: 6-206 months). Laterality was evenly distributed (right eyes: *n* = 67, left eyes: *n* = 57; *p* = 0.369). Lens status differed significantly at baseline (*p* < 0.001) and changed significantly during the follow-up, with pseudophakia increasing from 21 eyes (16.9%) at baseline to 43 eyes (34.7%) at final examination (*p* < 0.001).

### Visual acuity outcomes

Mean BCVA remained largely stable over the follow-up period. Mean BCVA was 0.16 ± 0.15 logMAR at baseline and 0.18 ± 0.15 logMAR at the final visit. The mean change in BCVA was +0.02 logMAR (95% confidence interval -0.01 to 0.04; *p* = 0.29), which was below the predefined threshold for a clinically meaningful change (≥ 0.1 logMAR). At the individual level, 44 eyes (35.5%) showed no relevant change (< 0.1 logMAR) in BCVA. Improvement of ≥0.1 logMAR was observed in 31 eyes (25.0%), including improvements of 0.1 logMAR in 19 eyes, 0.2 logMAR in 7 eyes, 0.3 logMAR in 1 eye, and ≥0.4 logMAR in 4 eyes. Worsening of ≥0.1 logMAR occurred in 49 eyes (39.5%), most commonly by 0.1 logMAR (29 eyes), followed by 0.2 logMAR (13 eyes) and 0.3 logMAR (7 eyes); no eye worsened by ≥0.4 logMAR. Thus, while individual eyes exhibited improvement or decline, the overall distribution was balanced, resulting in a small net mean change.

### Lens status and predictors of BCVA change

Stratification of BCVA change by lens status trajectory showed that eyes undergoing cataract surgery exhibited a significant improvement in BCVA (mean change in BCVA -0.08 ± 0.23 logMAR) compared with eyes that remained phakic ( + 0.04 ± 0.14 logMAR; *p* = 0.006). Eyes that were pseudophakic throughout follow-up showed minimal change ( + 0.01 ± 0.13 logMAR).

In univariable linear regression analyses, worse baseline BCVA was the strongest predictor for both greater improvement in BCVA over follow-up (ΔBCVA) and worse final BCVA (both *p* < 0.001). Cataract surgery during follow-up was associated with greater BCVA improvement and better final BCVA, whereas age, sex, follow-up duration, baseline CFT, schisis diameter, and other baseline OCT features were not significantly associated with either outcome. Subfoveal EZ disruption and final ERM-F subtype were associated with worse final BCVA in univariable analysis, with ERM-F subtype showing a significant global effect.

In multivariable linear regression analysis, worse baseline BCVA was independently associated with both worse final BCVA and with greater BCVA improvement over follow-up (ΔBCVA) (both *p* < 0.001). Cataract surgery during follow-up was independently associated with better final BCVA (*p* = 0.020) and greater BCVA improvement (*p* = 0.014). Final EZ disruption was not independently associated with either final BCVA (*p* = 0.145) or ΔBCVA (*p* = 0.274). Model performance was modest, with an adjusted R² of 0.22 for both models (Supplementary Table S1).

### BCVA according to ERM-F subtype

Baseline BCVA differed significantly among ERM-F subtypes (overall p = 0.016). Eyes with open-flat (0.14 ± 0.14 logMAR) showed better baseline BCVA compared with closed subtypes (0.20 ± 0.11 logMAR; *p* = 0.045). Differences between open-flat and open-elevated (0.20 ± 0.18 logMAR; *p* = 0.120) and between open-elevated and closed subtypes (*p* = 1.000) were not statistically significant.

Final BCVA also differed across final subtype groups (*p* = 0.006). Mean final BCVA was lowest in open-flat ERM-F (0.13 ± 0.13 logMAR) and highest in eyes with closed morphology (0.29 ± 0.18 logMAR) and LMH (0.33 ± 0.15 logMAR). Post-hoc Dunn testing identified a significant difference only between open-flat and closed subtypes (*p* = 0.035). Other pairwise comparisons were not significant (*p* = 0.11-1.00). Subgroup sizes were small for some categories (normal fovea, *n* = 2; LMH, *n* = 3).

Changes in BCVA (ΔBCVA) did not differ significantly between baseline subtypes (p = 0.275). Mean ΔBCVA was 0.06 ± 0.14 logMAR in closed eyes, -0.02 ± 0.21 logMAR in open-elevated eyes, and +0.02 ± 0.14 logMAR in open-flat eyes, with no significant pairwise differences (*p* = 0.42-0.91).

Among eyes that progressed to LMH (3/124), mean BCVA was mostly unchanged (baseline: 0.30 ± 0.12 logMAR; final: 0.33 ± 0.15 logMAR). In eyes developing a mixed ERM-F/LMH phenotype (14/124), BCVA remained essentially unchanged (baseline: 0.16 ± 0.15 logMAR; final: 0.16 ± 0.12 logMAR).

### Morphological features and subtype evolution

Intergrader agreement for ERM-F subtype classification was high at baseline, with concordant grading in 110 of 124 eyes (88.7%) and a Cohen’s κ of 0.82 (95% CI, 0.72-0.91). At the final visit, agreement remained high despite increased diagnostic complexity, with concordance in 115 eyes (92.7%) and κ = 0.89 (95% CI, 0.82-0.96). All discrepancies were resolved by consensus.

**Fig. 2:**
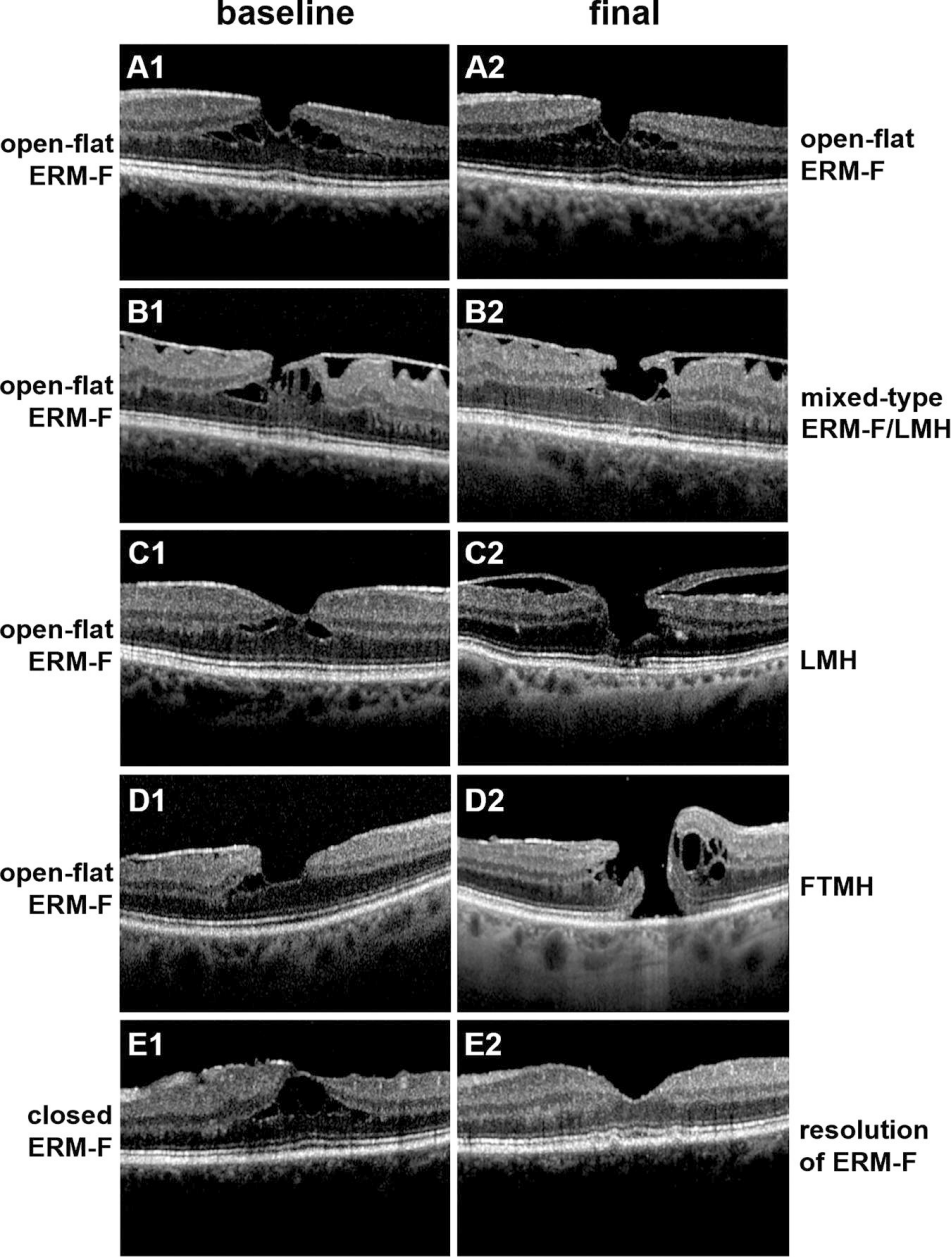
Representative horizontal Optical coherence tomography (OCT) B-scan images showing morphological evolution of various subtypes of epiretinal membrane-foveoschisis (ERM-F) from baseline to final follow-up. Each row displays an example case at baseline (left column) and final examination (right column). **A1, A2** Open-flat ERM-F configuration remains stable over time without morphological progression. **B1, B2** ERM-F open-flat at baseline evolves into a mixed-type ERM-F/LMH configuration with partial ERM-F regression and features (foveal contour irregularity, undermined foveal edge) of lamellar macular hole (LMH). **C1, C2** ERM-F open-flat progresses to LMH, with evident inner retinal tissue loss and cavitation. **D1, D2** Open-flat ERM-F developed into mixed ERM-F/LMH and subsequently progressed to full-thickness macular hole (FTMH) in two cases. **E1, E2** Closed ERM-F configuration at baseline shows near-complete resolution of tractional features and retinal schisis at follow-up.

At baseline, open-flat ERM-F was the most common subtype (61.3%, 76/124), followed by open-elevated (24.2%, 30/124) and closed ERM-F (14.5%, 18/124). Subtype evolution differed significantly according to baseline configuration, indicating substantial morphological reorganisation over time (*p* < 0.001; Fig. 2).

Among eyes with baseline open-flat ERM-F, the majority remained open-flat at final follow-up (50/76 eyes, 65.8%). Transitions most commonly occurred toward mixed ERM-F/LMH (11/76 eyes, 14.5%) or open-elevated ERM-F (10/76 eyes, 13.2%), while progression to LMH was seen in three eyes (3/76 eyes, 3.9%). Two cases converted to closed ERM-F (2/76 eyes, 2.6%).

Open-elevated ERM-F showed relative stability: 80.0% (24/30) remained open-elevated, five eyes (16.7%) converted to open-flat ERM-F, and one eye (3.3%) developed mixed ERM-F/LMH.

Closed ERM-F demonstrated the greatest heterogeneity. While 55.6% (10/18 eyes) remained closed, others transitioned to open-flat (3/18 eyes, 16.7%), reverted to normal foveal morphology (2/18 eyes, 11.1%), or developed mixed ERM-F/LMH (2/18 eyes, 11.1%).

**Fig. 3:**
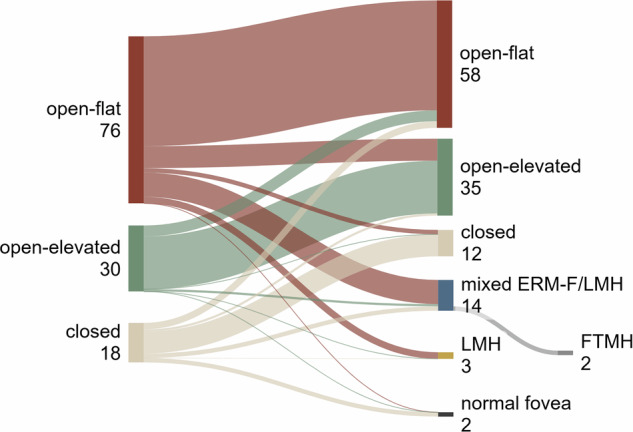
Sankey diagram illustrating the transition of retinal phenotypes from baseline to final assessment. The left column represents the baseline epiretinal membrane-foveoschisis (ERM-F) subtypes: open-flat (76/124 eyes), open-elevated (30/124 eyes), and closed (18/124 eyes). The right column displays the final phenotypes: open-flat (58/124 eyes), open-elevated (35/124 eyes), closed (12/124 eyes), mixed ERM-F/lamellar macular hole (LMH) (14/124 eyes), LMH (3/124 eyes), and normal foveal morphology (2/124 eyes). Two eyes progressed from open-flat via mixed ERM-F/LMH to full-thickness macular hole (FTMH) during follow-up; FTMH is shown as a separate terminal branch for illustration but was treated as a censoring event and not included in final subtype counts. The flow widths between baseline and final subtypes are proportional to the number of eyes transitioning between phenotypes, highlighting changes and stability in retinal conditions over time. Figure created using SankeyMATIC (sankeymatic.com).

Overall, mixed ERM-F/LMH developed in 11.3% (14/124 eyes), and 2.4% (3/124 eyes) progressed to LMH. In addition, two eyes (1.6%) that were open-flat at baseline progressed beyond mixed ERM-F/LMH to FTMH. These eyes were censored at the last pre-FTMH visit for ERM-F outcome analyses and were reported descriptively. At the final follow-up, open-flat ERM-F remained the most frequent phenotype (58/124 eyes, 46.8%), followed by open-elevated (35/124 eyes, 28.2%) and closed ERM-F (12/124 eyes, 9.7%). Normal foveal morphology without ERM-F or LMH features was observed in 1.6% of eyes (2/124) . Table 1 and Fig. 3 show subtype distributions at baseline and final visit.

**Table 1:** Morphological transitions of epiretinal membrane-foveoschisis (ERM-F) subtypes from baseline to final examination.

Baseline classifications include open (open-flat, open-elevated) and closed configurations. Over time, some eyes transitioned to other ERM-F subtypes, mixed ERM-F/lamellar macular hole (LMH), or LMH, or regressed to a normal foveal morphology; however, most eyes did not undergo morphological subtype transition. Two eyes progressed from open-flat ERM-F via a mixed ERM-F/LMH phenotype to full-thickness macular hole (FTMH) during follow-up and were censored at the last pre-FTMH visit; therefore, FTMH is not included as a final subtype. Data are shown as number of eyes and percentage of total number of eyes (*n* (%)).

### Quantitative and qualitative OCT features

Mean CFT remained stable (baseline: 217 ± 134 µm, median 186 µm, IQR 170-216 µm; final follow-up: 210 ± 77 µm, median 190 µm, IQR 166-228 µm; p = 0.457). At baseline, CFT differed significantly by ERM-F subtypes (*p* = 0.001). Eyes with closed ERM-F showed the greatest CFT (297 ± 111 µm), compared with both open-flat (208 ± 155 µm; *p* = 0.001) and open-elevated (191 ± 40 µm; *p* = 0.005) subtypes, while no difference was observed between open subtypes (*p* = 1.000). Similar differences persisted at final follow-up (*p* < 0.001). Closed morphology remained associated with the greatest CFT (304 ± 96 µm). Lower CFT values were measured in open-flat (209 ± 73 µm; *p* = 0.024), open-elevated (203 ± 57 µm; *p* = 0.024), mixed ERM-F/LMH (168 ± 54 µm; *p* < 0.001), LMH (190 ± 90 µm), and normal foveal morphology (127 ± 51 µm; *p* = 0.027). No significant differences were observed among non-closed subtypes (*p* = 0.063–1.000).

All eyes fulfilled the consensus criteria for ERM-F at baseline [1]. Mean horizontal schisis diameter remained stable at the cohort level (baseline: 1512 ± 651 µm; final: 1509 ± 1022 µm; *p* = 0.065), but individual changes were common: ≥10% increase in 34 eyes (27.4%), ≥10% decrease in 53 eyes (42.7%), and stability (< 10% change) in 37 eyes (29.8%). Schisis diameter change was not associated with ERM-F subtype (*p* = 0.916).

Overall, retinal wrinkling prevalence remained stable (baseline: 92/124 eyes, 74.2%; final: 95/124 eyes, 76.6%; *p* = 0.71) with bidirectional changes. Thirteen eyes showed resolution, while 15 eyes developed new wrinkling during follow-up. INL microcystoid spaces showed unchanged prevalence at baseline and final follow-up (46/124, 37.1%; *p* = 0.86), but dynamic individual transitions (resolution in 17 eyes, newly developed in 17 eyes). Foveal wall verticalisation increased significantly from 44 eyes (35.5%) to 56 eyes (45.2%) (*p* = 0.029). Among eyes without verticalisation at baseline, 21 developed this feature during follow-up, whereas 9 eyes showed resolution.

Undermined foveal edges and retinal tissue loss were absent at baseline but developed in 18 (14.5%) and 13 eyes (10.5%), respectively. Foveal base irregularity increased from three eyes (2.4%) to 18 eyes (14.5%; *p* = 0.036). A foveal bump newly developed in six eyes (4.8%). EP increased from nine eyes (7.3%) to 18 eyes (14.5%; *p* = 0.013). Subfoveal EZ disruption increased from 12 eyes (9.7%) at baseline to 18 eyes (14.5%) at final follow-up (*p* = 0.109), while four eyes showed partial restoration. EZ disruption was associated with worse BCVA, particularly at final follow-up. Morphological parameters are summarised in Table 2.

**Table 2:** Baseline and final values of quantitative and qualitative retinal parameters.

Variable	Baseline	Final	*P*-value
**Central foveal thickness, µm, mean ± SD**	217 ± 134	210 ± 77	0.457^†^
**Horizontal schisis diameter, µm, mean ± SD**	1512 ± 651	1509 ± 1022	0.065^†^
**Inner nuclear layer microcystoid spaces, n (%)**	46 (37.1)	46 (37.1)	0.860^‡^
**Retinal wrinkling, n (%)**	92 (74.2)	95 (76.6)	0.710^‡^
**Foveal wall verticalisation, n (%)**	44 (35.5)	56 (45.2)	**0.029**^‡^
**Undermined edges, n (%)**	0 (0)	18 (14.5)	**< 0.001**^**#**^
**Loss of retinal tissue, n (%)**	0 (0)	13 (10.5)	**< 0.001**^**#**^
**Foveal bump, n (%)**	0 (0)	6 (4.8)	**< 0.001**^#^
**Epiretinal proliferation, n (%)**	9 (7.3)	18 (14.5)	**0.013**^**‡**^
**Ellipsoid zone (EZ) disruption, n (%)**	12 (9.7)	18 (14.5)	**0.109**^‡^

^†^ Wilcoxon signed-rank test.

^‡^ McNemar test.

^#^ Exact McNemar test.

Values for continuous variables are expressed as mean ± standard deviation (SD), and categorical variables are presented as n (% of eyes). *P*-values were calculated using: Wilcoxon signed-rank test (†) for continuous data, McNemar test (‡) for paired categorical data, and exact McNemar test (#) for binary outcomes with zero baseline occurrence. Percentages are calculated based on the total number of eyes (*n* = 124).

Taken together, ERM-F eyes demonstrated morphological plasticity, characterised by dynamic remodelling of tractional features and progressive emergence of LMH-associated criteria, despite largely stable mean CFT and BCVA at the cohort level.

## Discussion

This study provides a comprehensive longitudinal analysis of treatment-naïve idiopathic ERM-F with a mean follow-up of more than four years. Despite significant morphological remodelling over time, mean BCVA remained largely unchanged at the population level, indicating that structural remodelling in ERM-F does not necessarily translate into loss of visual acuity.

At first glance, the largely unchanged mean BCVA in our cohort could be perceived as discrepant with a Japanese longitudinal study, which reported significant worsening of BCVA, metamorphopsia, and traction parameters in ERM-F eyes with enlargement of the foveoschitic area [3]. However, their data also demonstrated stability in eyes without foveoschisis enlargement, showing the heterogeneity of ERM-F. Our larger cohort includes a high proportion of eyes with unchanged function and morphology, alongside a minority with progressive remodelling towards LMH or mixed ERM-F/LMH. Furthermore, we only assessed BCVA, whereas Matoba et al. additionally quantified metamorphopsia (M-CHARTS) and traction indices, which may be more sensitive to subtle tractional progression than visual acuity testing. Accordingly, stable mean BCVA in our cohort does not preclude functional changes in other visual domains. Also, individual-level analyses in our study demonstrated heterogeneous BCVA trajectories, with both clinically meaningful improvement and worsening occurring despite a small non-significant mean change. Finally, eyes with more pronounced symptoms or visual decline were more likely to undergo surgery and were censored at their last preoperative visit, which may have attenuated the apparent decline in mean BCVA among the remaining conservatively followed eyes. Thus, our findings and those of Matoba et al. are best interpreted as complementary: both support a model in which ERM-F exhibits diverse trajectories, with a subset of eyes showing traction-driven functional deterioration and others remaining functionally stable despite morphological plasticity.

The open-flat configuration was the most common subtype at both baseline and final follow-up, though nearly one-third of eyes transitioned to other subtypes (Table 1). Notably, a substantial number of open-flat eyes developed retinal tissue loss and progressed to mixed ERM-F/LMH or LMH. Despite these structural changes, mean BCVA remained stable, consistent with prior reports. Matoba et al. [3] reported minimal visual decline over extended follow-up in untreated ERM-F, with stability particularly noted in eyes without foveoschisis enlargement (18 eyes; follow-up period 45.0 ± 29.5 months) and only mild BCVA decline (0.03 ± 0.09 to 0.09 ± 0.11) in eyes with foveoschisis enlargement (17 eyes; follow-up period: 29.1 ± 27.0 months). Our study further showed that eyes with the open-elevated subtype had poorer baseline BCVA than open-flat eyes, potentially due to increased vertical traction or schitic retinal disruption. This supports the prognostic relevance of anatomical configuration. Interestingly, some eyes exhibited spontaneous structural improvement, with reversion to normal foveal morphology.

While subtype transitions were common, they did not consistently correlate with BCVA changes. This suggests that visual function may depend more on specific structural biomarkers, particularly EZ integrity, than on subtypes alone. EZ disruption was related to worse BCVA in unadjusted analyses, consistent with prior reports. However, this association weakened after accounting for baseline BCVA and cataract surgery, likely reflecting both confounding by baseline BCVA and lens status as well as the relatively low prevalence of EZ disruption in our cohort, which limits statistical power. Our findings align with previous studies emphasising the role of photoreceptor integrity in visual prognosis for ERM-F and LMH [2, 8]. Schisis diameter did not correlate with BCVA, reinforcing that not all structural distortions translate to functional impairment.

Mean CFT changed minimally, indicating that CFT may be unsuitable as a marker of progression in ERM-F. In contrast, qualitative features such as undermined foveal edges and foveal wall verticalisation showed more marked progression. The presence of undermined edges and retinal tissue loss – hallmark features of LMH – support the concept that ERM-F may represent an intermediate phenotype between classic idiopathic ERM and LMH [1, 3]. The development of a foveal bump in some patients likely reflects active vitreomacular interface remodelling, possibly related to evolving traction or early retinal layer instability [1].

Previous reports on untreated ERM-F describe a heterogeneous natural history, with many eyes remaining functionally stable over time while a subset progresses toward LMH-like configurations, sometimes accompanied by visual acuity decline [3]. The biological mechanisms underlying these divergent natural courses remain incompletely understood. While ERM-F is defined as a tractional disorder driven by tangential forces exerted by a contractile ERM, growing evidence suggests that secondary intraretinal responses, particularly glial activation and Müller cell-mediated remodelling, may substantially influence disease progression and structural plasticity. Experimental models and OCT-based biomechanical analyses indicate that chronic tangential traction deforms the inner retina and stretches Müller cells, which are critical for maintaining retinal architecture, force distribution, and metabolic homeostasis [10, 11]. Sustained mechanical stress may compromise Müller cell integrity, leading to intraretinal fluid dysregulation and the appearance of microcystoid spaces within the INL, a feature increasingly recognised in ERM-F and related disorders [12]. Histopathologic evidence supports this traction-gliosis interaction. In idiopathic ERMs, greater glial cell density and structural complexity correlate with advanced OCT stages and worse visual outcomes, indicating that gliosis contributes beyond purely mechanical effects [13–15]. Ultrastructural investigations show that ERM-F membranes contain  both contractile myofibroblasts and glial-derived cellular components, supporting the concept that ERM-F lies on the tractional end of a broader ERM-LMH disease spectrum while sharing features of glial-driven remodelling [16, 17]. OCT biomarkers, such as hyperreflective retinal spots may reflect associated intraretinal cellular responses across disease stages [15]. High-resolution OCT studies comparing ERM-F, LMH, and MPH show that microstructural integrity, particularly of the outer retina and EZ, is closely linked to visual function, indicating that functional outcomes depend not only on tractional configuration but also on the extent of intraretinal tissue response [2]. ERM-F-specific data further support this concept. Yang et al. [18] demonstrated that different ERM-F configurations are associated with distinct foveal microstructural alterations and visual outcomes, supporting the role of structural plasticity and cellular remodelling in visual prognosis beyond traction alone.

Within this framework, the emergence of LMH-related features and intraretinal microcystoid changes observed in a subset of eyes in our longitudinal cohort may not be attributable to traction alone but instead reflect secondary gliotic remodelling of the inner retina. Our study did not include histopathologic validation or quantitative OCT biomarkers of gliosis; therefore, this interpretation remains hypothetical. Future studies combining longitudinal ERM-F phenotyping with imaging markers and histopathologic correlation could may further clarify the relative contributions of mechanical traction and intraretinal cellular responses to ERM-F plasticity.

Another interesting phenomenon, the cause of which remains unclear and warrants further investigation, is the observed female predominance in the prevalence of ERM-F  [3–5, 8, 19–21].

Some limitations of this study require consideration in the interpretation of our results. First, the retrospective design and tertiary referral setting may introduce selection bias, potentially overrepresenting symptomatic or advanced cases. Cases with higher functional decline may have been selected for surgery and thus been censored during follow-up, potentially underestimating long-term visual deterioriation in the overall ERM-F population. Second, the diagnosis of asymptomatic ERM-F cases limits precise estimation of disease duration and thus restricts temporal analaysis. Baseline assessment represents first clinical detection or presentation at our institution rather than disease onset; the pre-baseline duration is unkown and likely heterogenous. Finally, functional outcomes were limited to BCVA and did not capture other relevant symptoms, such as metamorphopsia. This study’s strengths include a large cohort providing a robust dataset for analysing the natural history and progression patterns of ERM-F. The use of a standardised OCT-based classification system enhances diagnostic consistency and reduces observer bias. The relatively long mean follow-up period offers valuable longitudinal data. Furthermore, reliance on real-world clinical data reflects typical patient trajectories, supporting external validity of our findings. Detailed OCT analysis allowed detection of subtle morphological changes such as EZ disruption, which may serve as a marker of disease progression. Emphasising subtype-specific features advances understanding of ERM-F heterogeneity and may help guide surgical timing, although the optimal timing of pars plana vitrectomy and ERM peeling remains unclear [3, 4, 8, 19–21]. Whether early surgery in patients progressing toward LMH preserves retinal structure or stabilises vision needs to be subject to future studies.

## Conclusion

ERM-F is a heterogeneous and structurally dynamic vitreomacular condition, most often following an indolent course but with a subset demonstrating progressive remodelling toward degenerative phenotypes. Specific structural biomarkers – most notably EZ disruption and potential features of LMH such as undermined foveal edges – may signal an increased risk of visual decline. These findings support the need for an individualised management approach, with regular observation for most patients and consideration of earlier surgical intervention in those exhibiting high-risk morphological changes.

Supplemental material is available at Eye’s website.

## Summary

### What was known before:

- ERM-F is characterised by tractional retinal schisis with a range of morphological subtypes all resulting from an ERM.

### What this study adds:

- This study identifies OCT-based morphological changes over time, including the development of features characteristic of lamellar macular hole (LMH) and visual outcomes in treatment-naïve ERM-F.
- This study informs clinicians about the variable natural history of ERM-F and highlights morphological changes, aiding patient counselling and management.

## Supplementary information

Supplementary Figure S1 – Figure Legend

Supplementary Figure S1

Supplementary Table S1

## Data Availability

Most of the data generated or analysed during this study are included in this published article. Further data are not publicly available due to data privacy reasons but are available from the corresponding author on reasonable request.

## **Declarations**

Ethical guidelines were followed, obtaining approval from the Institutional Review Board (IRB) or Ethics Committee (project number: 177/2020BO2). Informed consent was waived due to the retrospective design and anonymised data.
