# Supplementary material for: Morphological plasticity and visual acuity in the natural course of epiretinal membrane-foveoschisis: A longitudinal OCT study
Source: Eye (Lond). 2026 Feb 17;40(6):789–96. doi: 10.1038/s41433-026-04304-8 (PMC13061954; doi:10.1038/s41433-026-04304-8)
Supplement: Supplementary file 1 — Supplementary Figure S1 – Figure Legend [file 41433_2026_4304_MOESM1_ESM.docx]

**Supplementary Figure S1 – Figure Legend**

Supplementary Figure S1. Representative examples of morphological phenotypes of epiretinal membrane foveoschisis (ERM-F), lamellar macular hole (LMH) and mixed ERM-F/LMH graded in this study. (A) Normal foveal contour. (B) Open-flat ERM-F. (C) Open-elevated ERM-F, with foveal edges elevated above the surrounding retinal plane (white arrowheads). (D) Closed ERM-F with epiretinal membrane covering the fovea (white arrowhead). (E) Mixed ERM-F/LMH showing schisis, undermined edge (dashed arrow), and irregular foveal contour. (F) LMH with undermined edges and retinal tissue loss (dashed arrows), irregular foveal contour, epiretinal proliferation (star), foveal bump (white arrow), and ellipsoid zone disruption (black arrowheads).
