# Supplementary figures and images for: Morphological plasticity and visual acuity in the natural course of epiretinal membrane-foveoschisis: A longitudinal OCT study
Source: Eye (Lond). 2026 Feb 17;40(6):789–96. doi: 10.1038/s41433-026-04304-8 (PMC13061954; doi:10.1038/s41433-026-04304-8)

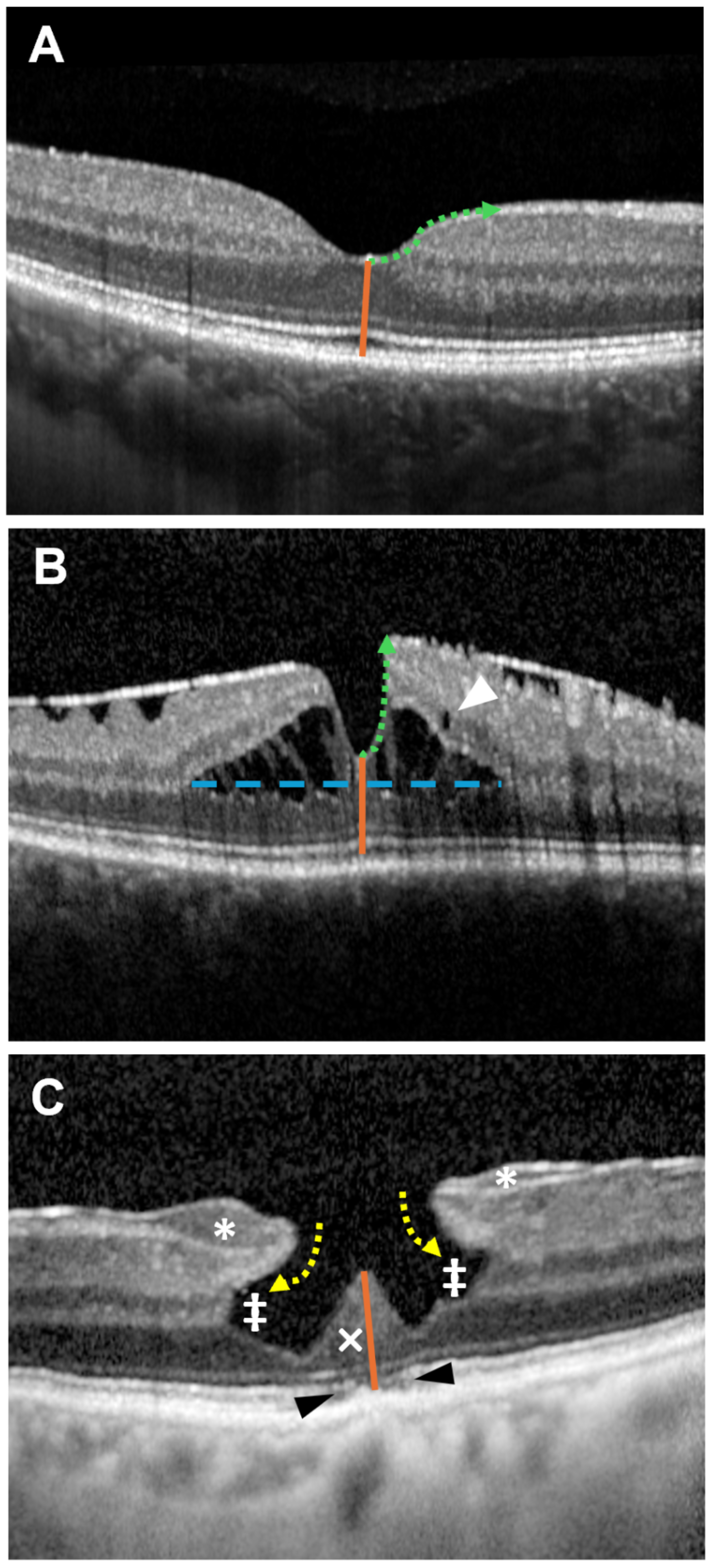

Supplement: Supplementary file 2 — Supplementary Figure S1 [file 41433_2026_4304_MOESM2_ESM.tif]
