# Supplementary material for: Morphological plasticity and visual acuity in the natural course of epiretinal membrane-foveoschisis: A longitudinal OCT study
Source: Eye (Lond). 2026 Feb 17;40(6):789–96. doi: 10.1038/s41433-026-04304-8 (PMC13061954; doi:10.1038/s41433-026-04304-8)
Supplement: Supplementary file 3 — Supplementary Table S1 [file 41433_2026_4304_MOESM3_ESM.docx]

**Supplementary Table S1**

| **Predictor** | **Final BCVA β (95% CI)** | **p value** | **ΔBCVA β (95% CI)** | **p value** |
| --- | --- | --- | --- | --- |
| Baseline BCVA (per 0.1 logMAR) | +0.045 (+0.028 to +0.062) | **<0.001** | +0.033 (+0.020 to +0.046) | **<0.001** |
| Cataract surgery during follow-up (yes/no) | -0.038 (-0.070 to -0.006) | **0.020** | -0.043 (-0.076 to -0.009) | **0.014** |
| Final ellipsoid zone disruption (present/absent) | +0.026 (-0.009 to +0.061) | 0.145 | +0.020 (-0.016 to +0.057) | 0.274 |
| Model performance: Final BCVA: adjusted R² = 0.22; ΔBCVA: adjusted R² = 0.22. | | | | |

**Supplementary Table S1 –** Multivariable linear regression models for final best-corrected visual acuity (BCVA) and change in BCVA (ΔBCVA). β coefficients represent the adjusted effect of each predictor on the dependent variable (logMAR). Baseline BCVA was rescaled and reported per 0.1 logMAR. Positive β values indicate worse visual acuity. Adjusted R² values describe model fit.
